# The Tolerance Model of Non-Inflammatory Immune Competence in Acute Pediatric Malnutrition: Origins, Evidence, Test of Fitness and Growth Potential

**DOI:** 10.3390/nu15234922

**Published:** 2023-11-25

**Authors:** Bill Woodward, Lyn M. Hillyer, Jennifer M. Monk

**Affiliations:** Department of Human Health and Nutritional Sciences, University of Guelph, Guelph, ON N1G 2W1, Canada; lhillyer@uoguelph.ca (L.M.H.); jmonk02@uoguelph.ca (J.M.M.)

**Keywords:** cytokines, severe acute malnutrition, immune tolerance, immune depression, interleukins, T cell, autoimmune disease, inflammation, endocrine hormones, adaptation

## Abstract

The tolerance model rests on the thesis of a physiologically regulated, albeit unsustainable, systemic attempt to adapt to the catabolic challenge posed by acute prepubescent malnutrition even in its severe forms. The model centers on the immunological component of the attempt, positing reorientation toward a non-inflammatory form of competence in place of the classic paradigm of immunological attrition and exhaustion. The foundation of the model was laid in 1990, and sixteen years later it was articulated formally on the basis of a body of evidence centered on T cell cytokines and interventions with cytokine and hormonal mediators. The benefit originally suggested was a reduced risk of autoimmune pathologies consequent to the catabolic release of self-antigens, hence the designation highlighting immune tolerance. Herein, the emergence of the tolerance model is traced from its roots in the recognition that acute malnutrition elicits an endocrine-based systemic adaptive attempt. Thereafter, the growth of the evidence base supporting the model is outlined, and its potential to shed new light on existing information is tested by application to the findings of a published clinical study of acutely malnourished children. Finally, some knowledge gaps pertinent to the model are identified and its potential for growth consonant with evolving perceptions of immunobiology is illustrated.

## 1. Introduction

Pediatric malnutrition is considered by the World Health Organization to include both overweight and underweight, the latter presenting in two main forms, viz. acute (wasting) discerned on the basis of weight-for-height and chronic (stunting) discerned on the basis of height-for-age [1]. The focus of attention herein is on acute types of prepubescent malnutrition because it was consideration of these manifestations that gave rise to the tolerance model hypothesis. The severity of acute pediatric malnutrition is determined on the basis of weight-for-height, mid-upper arm circumference and a clinical assessment regarding edema, and severe acute malnutrition manifests as marasmus (extreme emaciation), kwashiorkor (diagnostic feature being bilateral pitting edema without reference to anthropometric standards), or their combination, designated marasmic kwashiorkor [2,3]. According to the estimate produced by the World Health Organization for the year 2022 [1], 45 million children under five years of age suffer from acute malnutrition and close to 14 million of this number can be categorized as suffering severe acute malnutrition. As reviewed briefly elsewhere [4], all degrees of acute childhood malnutrition are associated, in a potently multiplicative relationship, with a heightened risk of morbidity and mortality from infection. In turn, a substantial body of preclinical and clinical research (e.g., [5,6]) is widely, even generally, interpreted to demonstrate that depressed inflammatory immune competence constitutes the physiological connection between acute malnutrition and susceptibility to infection. 

**Figure 1:**
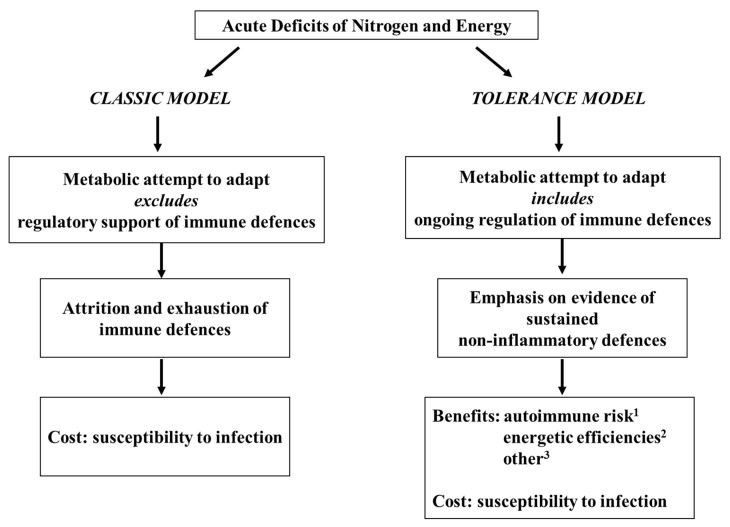
Classic model versus tolerance model of immune competence in acute pediatric malnutrition. ^1^ Section 2.3, Section 3.3, Section 3.4 and Section 6.1; ^2^ Section 4.5 and Section 6.2; ^3^ Section 3.4, Section 4.5, Section 6.2 and Section 6.4.

Since the inception of nutritional immunology as a distinct research front, unregulated attrition has been an implicit governing postulate with respect to the inflammatory immune depression characteristic of acute pediatric malnutrition [7]. A more sophisticated paradigm is needed to accommodate the gamut of experimental and clinical information, and a proposition formally designated the tolerance model in 2006 [8] has been offered for this purpose. The tolerance model posits that, as acute malnutrition progresses even into its advanced stages, immunological adaptability remains sufficient to permit the remodeling of immune defences toward a non-inflammatory form of competence. At this stage, the unifying potential of the tolerance model has been outlined vis-à-vis the prevalent, but antithetical, postulate of unregulated immunological debilitation [7,9], and the proposition has attracted some wider consideration [5,10]. In addition, key features of the model also have proven viable, and appear to be gaining traction, without reference to the larger paradigm. These features include, most notably, a tilt toward a non-inflammatory cytokine disposition [3,5,10,11,12,13,14,15,16,17,18] and, more broadly, that the immunological characteristics of acute forms of malnutrition are part of a physiologically regulated systemic response [10,11,19,20,21,22,23,24,25,26,27,28] that compounds the risk of infection but also bestows adaptive benefits of an immunological nature [11,26,28,29,30,31]. Figure 1 highlights the incompatibility in core essence between the classic paradigm of immunological exhaustion and the tolerance model with its focus on restructuring immune competence within the framework of a larger, systemic attempt to adapt to acute forms of pediatric malnutrition. 

The main purpose herein is to position the tolerance model within the purview of nutritional immunology by providing a realistic assessment of its evidence base and potential. This objective is addressed, first by tracing the origins of the model (Section 2 and Section 3), second by outlining the growing body of evidence consistent with the model and incompatible with the classic paradigm of immunological attrition and dysfunction (Section 4), third by subjecting the model to a test of its fitness to improve understanding of the existing evidence base (Section 5), fourth by identifying deficiencies in the evidence base of the model (Section 4.1, Section 6.1, Section 6.4 and Section 7), fifth by demonstrating its freedom from some potentially constrictive assumptions (Section 6.2, Section 6.3, Section 6.4) and sixth by illustrating the potential of the model to grow and develop in harmony with, and propelled by, its parent discipline of immunobiology (Section 6.5). 

A secondary purpose of this article is to demonstrate the need, pointed out elsewhere [3,7], for improved animal modeling in the study of acute childhood malnutrition. Regardless of opportunities for improvement of animal modeling, however, experimental malnutrition cannot be expected to replicate the constellation of concomitant micronutrient deficiencies, exposures to infectious microorganisms, social challenges and environmental influences, let alone the genetic heterogeneity, characteristic of acute malnutrition in childhood. At the same time, the importance of animal modeling is undeniable for generating hypotheses, for suggesting human clinical management possibilities and for providing proof-of-concept. In each of these several ways, an account of the research path leading to the articulation of the tolerance model illustrates the potential of a long-term commitment to the analysis of animal models validated in relation to critical features of acute pediatric malnutrition. In this connection, findings secured through a research program centered, ultimately for three decades, on the immunological analysis of two validated weanling rodent models provided a cohesive point of reference necessary to the development of the tolerance model from its earliest explicit but tentative moments [32,33] to its first formal articulation [8] and beyond [7,9,34,35,36,37,38,39,40]. 

## 2. First Steps (1950s–1990s): Origins of a Tentative Tolerance-Centered Proposition

The core theme of the tolerance model was first proposed thirty years ago [32,33] and remains unchanged [7,9,34], viz. that the immune depression characteristic of acute pediatric malnutrition is one component, only, of a larger systemic attempt to adapt. According to the model, as first conceived, the immunological component of the adaptive attempt manifests as a remodeling of immune defences toward a non-inflammatory form of competence, and a presumptive benefit, secured at the cost of susceptibility to infection, is a reduction in the risk of inflammatory autoimmune sequelae to a catabolic challenge. Additional possible benefits have been indicated in more recent years (Section 4.5, Section 6.2 and Section 6.4) and, with some clinical and experimental support, center on the conservation of limited substrate and energy reserves in the face of the costs of inflammation. In any case, the model, notably, proposes nothing more than an adaptive attempt which, without external support, is unsustainable. 

### 2.1. The Endocrine–Immune Nexus 

Endocrine hormonal characteristics were among the early points of interest in relation to the physiology of acute pediatric malnutrition. By the mid-1970s, the proposition was widely acknowledged that at least some malnutrition-associated endocrine characteristics represent adaptive attempts (e.g., [41,42,43]), and this level of understanding quickly developed to take the form of a coordinated and systemic attempt (e.g., [42,44,45,46]). Importantly, the concept of neuroendocrine-mediated adaptation continues to develop in breadth and sophistication (e.g., [47,48,49]), recently extending to unanticipated and seemingly anomalous endocrine hormonal actions in support of the proffered adaptive attempt [48]. In its earliest form, however, this adaptation-centered point of view took its shape from studies that invoked (without suggesting adaptive benefit) the high blood concentration of glucocorticoids, characteristic of acute malnutrition, as a contributor to the lymphoid atrophy and cell-mediated immune depression observed in this pathology. The first of these investigations was conducted using young adult rats [50], but subsequent investigations of children [51], weanling monkeys [52] and weanling rodents [52,53] placed attention on the prepubescent stage of life. 

The cited early reports could not establish causation specific to the glucocorticoids, but they facilitated the formation of a connection, conceptually, between the endocrine and immunological components of the pathophysiology of prepubescent malnutrition in its acute forms. This point of understanding is now frequently invoked within the discipline (e.g., [54]) and is widely respected, also within the broader field relating diet and immune defence competence [47,55]. Notably, the concept has grown to take the form of a response to nutrient quantity and balance that includes cytokines and neural mediators in addition to endocrine hormones [55]. 

By the latter half of the decade of the 1970s, the concept connecting hormonal mediators and malnutrition-associated immune defences had achieved sufficient momentum to appear in secondary sources [56,57], wherein endocrine candidates additional to the corticosteroids (notably epinephrine, insulin, growth hormone and the thyroid hormones) were named as possible players, albeit without experimental or other direct investigative evidence. This conceptual scaffolding, at the time more intriguing than compelling, prompted a series of investigations in which supplements of triiodothyronine were given to weanling mice subjected to either of two metabolically dissimilar forms of severe acute nitrogen and energy deficit. Regardless of the form of malnutrition, and despite advancing nutritional deficits, the hormonal intervention prevented depression in primary thymus-dependent and -independent antibody responses [58,59,60,61] and in at least one primary cell-mediated response [61], although it was ineffective against another [59]. Importantly, these outcomes pertaining to function manifested in the face of unabated and profound lymphoid atrophy; in fact, in two models of extreme nitrogen deficits [60,61], the intervention accelerated malnutrition-associated lymphoid involution. Through the lens of endocrine hormonal support, therefore, functional immune plasticity—incompatible with a model of unregulated debilitative attrition—was evident on the part of at least some adaptive defences despite advanced and ongoing catabolic malnutrition. Further investigation, therefore, could be encouraged from the standpoint of clinical prospects. 

### 2.2. The Earliest Intervention Studies 

The earliest intervention studies relevant to the tolerance model comprise a conglomerate of investigations that preceded the series outlined (Section 2.1) with respect to triiodothyronine and adaptive defences. These investigations are considered more fully elsewhere [32,62], but seven are noted here to illustrate the diversity within the evidence base. In the first of the seven investigations, adrenalectomy prevented lymphopenia in adult rats subjected to an acute dietary nitrogen deficit [50] and, twenty years later, the same surgical intervention prevented the involution of lymphoid organs when imposed on acutely protein-deficient weanling mice [53]. Five studies followed shortly thereafter and, in each case, the strategy of administering bioactive compounds was adopted in favour of a surgical intervention. In the first of these investigations, acutely malnourished rabbits generated a fever response, despite ongoing severe weight loss, when administered chemically undefined pyrogens derived from animals fed a complete diet [63]. Additional reports soon emerged of investigations in which rodents, despite various forms of severe acute malnutrition, exhibited immune defence capabilities when administered exogenous agents including the glucan lentinan [64], a purified preparation of interleukin (IL)-1 [65] and a chemically ill-defined extract containing one or more thymic hormones [66,67]. The response capabilities assessed included innate [63,64,65] and adaptive [66,67] defences as well as resistance to infectious disease challenges [64,65]. 

Only two of the cited investigations [53,64] addressed the weanling stage of life, although two other studies [63,65] were initiated with somewhat older prepubescent animals. Nevertheless, the two studies employing surgical intervention (adrenalectomy) [50,53] persuasively connected the endocrine and immunological consequences of acute malnutrition, although the findings were not rigorously specific to any particular hormonal candidate. Moreover, the five investigations that centered on a biochemical intervention were designed in a manner permitting discernment of immune restoration because, in each case, an intervention was initiated subsequently to the development of depression in the chosen index of inflammatory defence. Importantly, too, the immunological and disease resistance outcomes were assessed independently of any rehabilitative attempt and, in three studies of prepubescent animals [63,64,65], the response manifested within twenty-four hours or less. Taken together, therefore, the findings constitute a body of evidence that immunological plasticity is sustained into the advanced stages of acute malnutrition, perhaps even during prepubescence. This was a substantive contribution toward the earliest explicit expression of the tolerance model [32,33,62]. Moreover, the findings provided a segue to interventional studies using endocrine mediators, the series centered on triiodothyronine and adaptive defences (Section 2.1) being the first to follow. 

### 2.3. An Adaptive Attempt: Tolerance of Self-Antigens Enters the Discussion

The body of early intervention studies (Section 2.1 and Section 2.2), including evidence connecting endocrine and immunological characteristics of acute malnutrition (Section 2.1), provided a modest basis for inferring that malnutrition-associated inflammatory immune depression is best understood as part of a systemic attempt to adapt to acute deficits of nitrogen and energy. This inference was first introduced explicitly in a presentation to the XIV International Congress of Nutrition (Seoul, Republic of Korea, 20–25 August 1989) [62], then in journal format a few months thereafter [32], and in a second conference setting two years later [33]. In the same venues [32,33,62], it was noted that a regulated adaptive attempt includes, ipso facto, an expectation of benefit. This point of reasoning led to a suggestion, prompted by considerations put forward by others regarding major physical trauma [68,69], as to a possible benefit from the inflammatory immune depression of catabolic malnutrition [32,33]. Physical trauma characteristically features hypercortisolemia, and the associated depression of inflammatory competence was proposed to reduce the risk of autoimmune pathologies pursuant to cortisol-mediated catabolic release of self-antigens [68,69]. The hormonal and immunological similarities between physical trauma and acute forms of prepubescent malnutrition, therefore, formed the basis for extending the tolerance-centered conjecture regarding trauma to the setting of acute pediatric deficits of nitrogen and energy [32,33]. The suggestion was entirely speculative. Moreover, it was acknowledged that any accruing benefits would come at the cost of susceptibility to opportunistic infections [33]. 

It is important to interject, at this juncture, that the concept of hormonally-regulated immunological change in response to malnutrition was articulated independently by three additional research groups within the ensuing decade [20,21,22,23,24]. The spotlight centered particularly on leptin, which emerged as a candidate both to initiate “adaptation of the organism to starvation … by metabolic, endocrine, and immunological changes” [24] and to regulate the balance between inflammatory competence and risk of autoimmune disease [30]. In addition, plausible suggestions were offered as to benefits in terms of energy apportionment for preservation of vital functions [20,22]. It must be noted that the leptin-centered research front relied on an adult rodent starvation model of questionable relevance to acute prepubescent malnutrition. Nevertheless, pursuit of the leptin hypothesis introduced important new energy to the proposition that malnutrition-associated immune defences must be understood as part of a larger, regulated physiology. When first expressed [32,33,62], this proposition represented a substantive departure from the prevailing perspective in which immunological characteristics were considered essentially in isolation. More recently, however, the field of immunometabolism has embraced the realization that a mature understanding of immune defences places them within the purview of a physiological regulatory network [28,55,70]. 

## 3. The Tolerance Model Takes Shape (1990s–2006)

From its earliest days, the research effort regarding malnutrition-associated immune depression centered on the T cell system [4,6,7,32,33,56,57,71]. This focus originated with, and was sustained by, reports documenting the blood lymphocyte profile, the exquisite sensitivity of the thymus (“a barometer of malnutrition” [22]), the histopathology of secondary lymphoid organs and the particular vulnerability of the cell-mediated type of adaptive immune response. Therefore, the first research question to be addressed in relation to the nascent tolerance proposition [32,33] addressed a prediction that the T cell compartment would restructure in a manner consistent with reduced inflammatory propensity. 

### 3.1. T Cell Subset Balance Suggests Quiescence in Acute Pediatric Malnutrition

Balance in numbers between T cells and B cells and between CD4^+^ and CD8^+^ T cell sub-populations attracted early attention because of the type of information available from clinical studies [56,57]. These important possibilities, however, came under challenge through studies of weanling mice that permitted invasive investigations of the total recirculating pool of lymphocytes [72], the lymphocyte profile of secondary lymphoid compartments [72,73,74] and a revealing comparison of the latter with the blood T cell profile [74]. Consequently, beginning in the middle of the decade of the 1990s, investigations progressed to examination of subsets within the CD4^+^ and CD8^+^ sub-populations of T cells. Attention centered on a surface marker designated CD45RA because this protein identifies a quiescent, naïve-type subset with consequent stringent activation requirements. Thus, a series of studies using weanling mouse models revealed a particular abundance of CD45RA^+^ elements within CD4^+^ and CD8^+^ sub-populations both in secondary lymphoid organs wherein immune responses arise [73,75,76,77] and in the blood [75,76,77]. In concordance with the model, the super-abundance of quiescent-phenotype T cells appeared to be independent of high-level antigenic challenge and, hence, to be a fundamental characteristic of the T cell system in acute malnutrition [73]. Moreover, the phenomenon did not reflect a delay in immunologic ontogeny [73,75,76,77], and a regulated adaptive purpose was further implicated by evidence that involution of the CD4^+^ sub-population was confined to the effector/memory (CD45RA^−^) subset [76]. This series of investigations, although limited to experimental animals, provided the first intentional test of the nascent tolerance model. Importantly, a surface marker analysis of blood T cells from acutely malnourished children yielded corroborating findings soon thereafter, although interpretation was complicated somewhat by the factor of concurrent infection [78]. 

### 3.2. Intervention Studies

Intervention studies have provided the most persuasive evidence that immune defence plasticity is sustained into the advanced stages of acute malnutrition, even its severe prepubescent forms. Investigations of this type, outlined in Section 2.1 and Section 2.2, were foundational to the first primitive formulation of the tolerance model hypothesis [32,33] and, subsequently, necessary momentum leading to the formal expression of the paradigm in 2006 [8] was provided by eight additional investigations of interventional design. 

Five investigations extended the database, revealing plasticity among innate defences. Triiodothyronine intervention prevented depression in natural killer cytotoxic activity in two mouse models of acute weanling malnutrition [79] and, in concurrent work, three additional interventions centered on the macrophage [80,81,82]. Thus, in an adult mouse model of severe nitrogen deficit, granulocyte-macrophage colony-stimulating factor restored the ability of macrophages to produce IL-6, superoxide and nitric oxide and, importantly, also restored resistance to a yeast infection [80]. In other work, injections of macrophage colony-stimulating factor restored the development and numbers of Kupffer cells, albeit with no reported index of function, when administered to weanling mice subjected to a catabolic nitrogen deficit [82]. Likewise, a glucocorticoid hormone receptor antagonist (mifepristone) eliminated depression in the capacity of peritoneal macrophages to produce superoxide and IL-6 following stimulation in vitro when administered throughout the progression of a catabolic nitrogen deficit in an adult mouse model [81]. In this investigation, adrenalectomy effected the same outcome, but the interpretation of the report hinges primarily on the pharmacological specificity of the chemical intervention. This report provides probably the most forceful single piece of evidence implicating high concentrations of glucocorticoids in relation to the immunological characteristics of acute forms of malnutrition. Finally, in a fifth investigation, a probiotic intervention concurrent with severe dietary restriction invigorated the intraperitoneal influx of neutrophils on the part of young adult mice in response to a sterile irritant [83]. The response to the intervention also manifested in terms of the production of several cytokines, viz. IL-6, IL-10 and macrophage inflammatory protein-2, by cells recovered from peritoneal exudates. Interpretation of this interesting study is hampered by the absence of a positive control; nevertheless, immune plasticity was evident in an advanced stage of ongoing catabolic malnutrition. 

During this interval of time, a research front centered on leptin further extended the list of effective intervention mediators. Injections of leptin during a two-day starvation period enabled adult mice to generate a vigorous cell-mediated response, which was almost undetectable in the absence of the hormonal support [20]. Soon thereafter, leptin interventions, using the same experimental system, were reported to attenuate splenic and thymic lymphoid involution [21] and to sustain a balanced response to bacterial endotoxin on the part of the inflammatory cytokines, tumour necrosis factor (TNF)-α and interferon (INF)-γ [23]. The relevance of this type of short-term, adult starvation model to prepubescent forms of acute malnutrition is unclear. Broadly, however, the findings contributed to the growing evidence base pointing to persistent endocrine hormonal control over immune defence capacities despite severe, catabolic malnutrition. 

Collectively, the eight cited reports afforded a new breadth of support to the proposition that immune defences, both innate and adaptive, retain responsiveness to physiological mediators in advanced stages of acute malnutrition—perhaps even during prepubescence [79,82]. Particularly forceful evidence of sustained immunological adaptability derives from the two studies [80,82] in which intervention was initiated at an advanced stage of malnutrition and the response was achieved in the face of unabated weight loss. Further, one of the latter two studies addressed the weanling stage of life [82] and, yet, the malnourished animals were able to respond to intervention within a matter of hours. 

### 3.3. Cytokine Production and Blood Concentration Profiles: The Tolerance Model Formalized 

By the beginning of the new century, an evidence base was established pointing to an emphasis on T cell quiescence in association with acute pediatric deficits of nitrogen and energy (Section 3.1). In turn, this small body of information directed attention to the network of cytokines that determine T cell characteristics and the global disposition of the T cell system. 

A small body of experimental and clinical evidence produced in the decade of the 1990s [80,84,85,86,87] prompted a tentative suggestion to the 45th Nestle Nutrition Workshop (Bangkok, Thailand, 29 March–1 April 1999), that research should be directed toward the possibility that acute forms of malnutrition alter “the balance between Th1- and Th2-type cytokines” [6]. Shortly thereafter, information relevant to this proposition was provided by a piece of work in which T cell mitogen-stimulated blood mononuclear cells of moderately wasted infants (weight-for-height z score, −1.3) produced more IL-4 and less INF-γ when first admitted for clinical care than following stabilization [88]. Although T cell counts were not included in the report, this minor omission was tempered by the assessment of complementary cytokines. Within three years, a second study of a cytokine panel conferred a modicum of additional momentum to the proposition regarding T cell cytokine balance. In this investigation, an elevated expression of type 2 cytokines (IL-4 and IL-10) was reported together with diminished expression of inflammatory type 1 mediators (IL-2 and INF-γ) by T cells from blood samples of acutely malnourished children [89]. The index applied in this instance was the percentage of T cells exhibiting intracellular cytokine expression. Notably, this report addressed constitutive cytokine expression, arguably revealing a baseline characteristic of the T cell compartment. By contrast, preceding investigations [80,84,87,88] centered on T cell cytokine production elicited by either antigens or mitogens. Studies of humans are of obvious importance but are limited to the blood compartment, which may be unrepresentative of the sites wherein immune responses arise [90]. It is worthy of note, therefore, that the findings pertaining to the cytokine profile of human blood T cells [88,89] are consistent with preceding animal-based reports [80,84,87] pertaining to T cells from secondary lymphoid organs. 

Although easily overinterpreted, blood cytokine concentrations represent spillovers from extravascular sites and can be viewed as reflecting, while not representing, concentrations at extravascular sites of action [91]. Arguably, therefore, assessment of blood cytokine concentrations provides a more broadly based view of systemic immunological character than can be gleaned from studies confined to cytokine-producing elements. Two reports pertaining to blood cytokine concentrations [85,92] were particularly influential, leading to the suggestion [6] that acute malnutrition tilts the balance of cytokines in a non-inflammatory direction. In the first place, elevated concentrations of IL-4 were found in blood samples taken from acutely malnourished children in a study that appeared to eliminate the confounder of nematode worm infestation [85]. Subsequently, high blood levels of transforming growth factor (TGF)-β, a potent anti-inflammatory and tolerizing cytokine, were reported in acutely malnourished prepubescent guinea pigs following exposure to live tuberculosis organisms and challenge with tuberculin [92]. 

As the body of information grew, the proposition was expanded to include cytokine-producing elements other than T cells and was refined to place emphasis on the potently anti-inflammatory and tolerogenic cytokine, IL-10 [93]. The evidence leading to this conceptual development included reports regarding macrophage cytokine production in vitro [80,83], blood concentrations of IL-10 (at that time limited to conference presentations) and blood concentrations of macrophage-derived inflammatory cytokines [23]. Reports pertaining to blood levels of IgE, characteristically high in acutely malnourished children [57], also were influential. In particular, although IgE is a quintessential Th2 class of antibody [94], its high blood levels emerged as independent of nematode worm infestation both in a cohort of acutely malnourished children [85] and in two forms of acute weanling rodent malnutrition [93]. Hence, a fundamental Th2-type disposition may be inferred. Notably, high blood levels of IgE did not reflect a mature, specific immune defence capability [85]—a most important point emphasized again more recently [95]. The proposition of type 2 and anti-inflammatory cytokine partiality in the context of acute pediatric malnutrition [6,93] neither includes nor requires any suggestion of corresponding anti-infectious defence competence. 

Blood cytokine levels are particularly informative when either complementary or opposing cytokines are considered together. This reasoning gave rise to an investigation of the blood concentrations of IL-10 and TGF-β in weanling mice subjected to acute forms of nitrogen and energy deficit [8]. These cytokines were selected as the dominant cytokine mediators of peripheral tolerance, and their blood bioactivities were high in two metabolically distinct forms of acute malnutrition [8]. Importantly, this outcome was not attributable simply to a delay in ontogeny that might attend a wasting disease at the weanling stage of life. Notably, also, the β1 isoform—the prepotent, perhaps exclusive, immunoregulatory member of the TGF-β family [96]—accounted substantially for the 20-fold elevation in blood TGF-β bioactivity which was found in both forms of acute weanling malnutrition [8]. 

**Figure 2:**
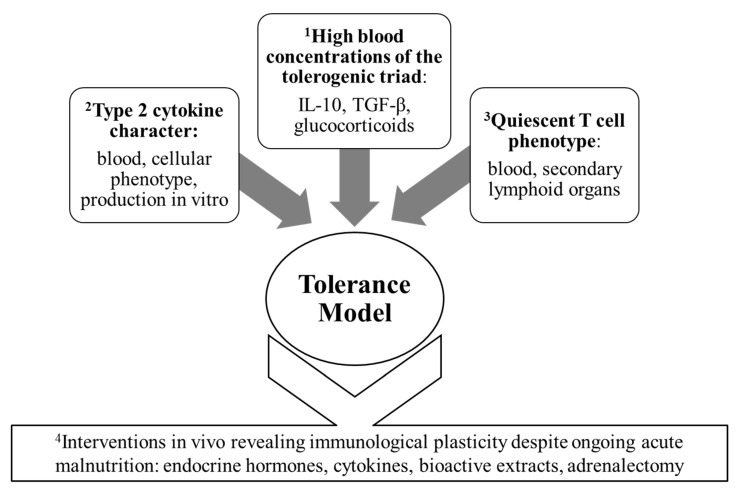
The four main clusters of immunological evidence leading to the formal articulation of the tolerance model. ^1^ Section 2.1 and Section 3.3; ^2^ Section 3.3; ^3^ Section 3.1; ^4^ Section 2.1, Section 2.2 and Section 3.2.

Prompted by the perception that the blood cytokine profile reflects a systemic immunological character, the “tolerance model” was articulated in 2006 [8] to accommodate the growing body of reports collectively suggesting that immunologic adaptability and a predominantly non-inflammatory form of immune competence are sustained into advanced stages of acute prepubescent malnutrition. This synthesis was strengthened by the recognition that glucocorticoid hormones, well known to be found at high levels in the blood of acutely malnourished children and prepubescent animals [42,43,44,45,46,51,52,53,97], would combine with IL-10 and TGF-β to comprise a potent triad of tolerogenic mediators [8]. Of some note, high blood concentrations of the three mediators manifested together in advanced stages of acute malnutrition in each of two metabolically dissimilar weanling mouse models [8,97], and this finding played a significant role in pointing to a probable functional unit of blood tolerogens. Figure 2 illustrates the distinct importance of the studies of interventional design while highlighting the four main threads of evidence that, collectively, prompted the formal expression of the tolerance model. 

### 3.4. Putative Benefits of Inflammatory Immune Depression: Newer Proposals 

During this period, the possibility that benefits could accompany malnutrition-associated immune depression was put forward by two additional research teams [29,30]. The proposals were made independently of one another and of the tentative original proposition [32,33]; moreover, they preceded the explicit articulation of the tolerance model [8]. One group suggested a reduction in the risk of infection-related granulomatous complications [29] and, subsequently, the second group [30] tendered a thesis proposing leptin to be the governing hormonal factor in achieving a balance between inflammatory immune competence and the risk of autoimmune pathologies. According to the “leptin hypothesis” [30], low concentrations of this hormone contribute decisively to the cell-mediated immune depression of malnutrition while, as an incidental benefit, reducing the risk of inflammatory autoimmune disease. In effect, therefore, the hypothesis offered a physiological underpinning for the original speculation [32,33] regarding protection against autoimmune pathologies. Some experimental evidence was cited in support of the hypothesis [30], notably (for present purposes), studies of leptin intervention in an adult rodent starvation model (Section 3.2) and an investigation in which caloric restriction was imposed on rodents susceptible genetically to a form of autoimmune disease (further comment in Section 6.1). 

It is important to recognize the additional momentum contributed (to the idea that inflammatory immune depression might confer benefits) by these two independent propositions. It is also important to recognize, however, that the propositions [29,30] differed substantially from the original suggestion [32,33] insofar as each represented malnutrition-associated immune depression as primarily an impairment, and the suggested benefits were portrayed as incidental, even accidental. By contrast, the nascent tolerance proposition [32,33] was framed to emphasize physiologically intentional benefits, while recognizing the attendant cost of susceptibility to opportunistic infection—an ultimately unsustainable survival strategy. This theme remained at the core of the proposition when, as outlined in Section 3.3 herein, it was formally designated the “tolerance model” in 2006 [8]. 

## 4. Ongoing Development of an Evidence Base Consistent with the Tolerance Model (2007 to the Present)

**Figure 3:**
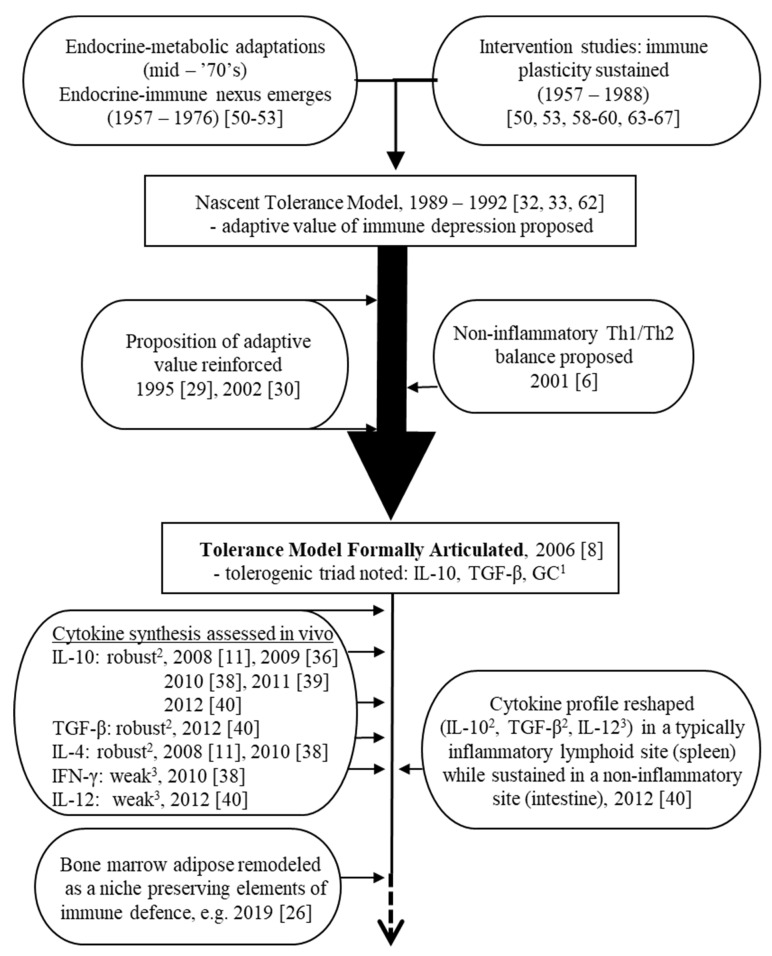
Timeline showing critical milestones in the continuing development of the tolerance model. ^1^ GC: glucocorticoid hormones; ^2^ production by malnourished either not different from, or greater than, well-nourished; ^3^ production by malnourished less than well-nourished. [6,11,26,29,30,32,33,36,38,39,40,50,51,52,53,58,59,60,62,63,64,65,66,67].

Several lines of evidence can be cited in the development of the platform for the tolerance model following its articulation in 2006. Broadly, these threads can be categorized as either primarily immunological in nature (i.e., centered on the cytokine profile or on the responsiveness of immune defences to intervention attempts), or primarily metabolic in nature. These ongoing research fronts serve both to uphold the model and to highlight potential growth points. A timeline is shown in Figure 3 highlighting milestones in the development of the tolerance model before and after its formal expression in 2006.

### 4.1. Blood Cytokine Concentrations 

The reports regarding constitutive blood cytokine concentrations that prompted the articulation of the tolerance model proposition (Section 3.3) focused on type 2 and anti-inflammatory mediators to the exclusion of type 1 cytokines. An early direct test of the formalized proposal, therefore, centered on INF-γ [35]. In this piece of work, low constitutive blood bioactivities of INF-γ were found in both forms of acute weanling malnutrition that had been reported, in the previous year [8], to elicit high bioactivities of IL-10 and TGF-β. Moreover, as reported in relation to these non-inflammatory, tolerizing cytokines [8], the outcome pertaining to INF-γ was not attributable simply to a delay in ontogeny. Importantly, this animal-based investigation was soon corroborated and extended by reports pertaining to constitutive cytokine concentrations in the blood of acutely malnourished children, viz. low concentrations of INF-γ [98], IL-2 [98,99] and IL-12 [12,98] coupled either with high concentrations of IL-10 [99] or, at least, with levels of this cytokine that were apparently unaffected despite catabolic malnutrition [12]. 

In the meantime, young adult mice subjected to a wasting dietary nitrogen deficit were reported to exhibit low blood type 1 cytokine immunoactivities [31] but high blood IL-10 immunoactivity [11] in response to an inflammatory stimulus. A model of adult malnutrition may be of uncertain relevance to the prepubescent stage of life, but the consonant responses in terms of types 1 and 2 cytokines are noteworthy. Moreover, these findings could be interpreted as evidence pertaining to systemic production and/or turnover. In addition, one of the reports [11] included the important interpretation that the finding in relation to IL-10 might reflect “an initial adaptation” and could represent “a mechanism to control the inflammatory response in…malnourishment.” Coming only two years after the formalization of the tolerance model [8], this provided important independent support for a core aspect of the model, viz. an adaptive attempt leading to a pre-determined benefit. 

During the same interval of years, additional studies [36,37] confirmed high blood bioactivities of IL-10 and TGF-β in the advanced stages of acute weanling malnutrition and also provided evidence that the blood levels of these tolerogenic cytokines rose early in the progression of weight loss. These findings dovetailed with a similar outcome pertaining to the blood concentration of corticosterone in the same weanling animal systems [97]. In view of the possible biphasic influence of glucocorticoids on inflammation, stimulatory at low concentrations and inhibitory at high levels [100], the reported critical illness level of elevation in corticosterone concentration [97] assumes particular significance. Of some note, the magnitude of the response, which remains worthy of research attention, came to light because of the availability and application of an exsanguination technique [101] that minimizes the impact of pre-anesthesia stress. In sum, IL-10, TGF-β and the glucocorticoid hormones—previously submitted as a triadic unit upholding adaptive attempts during advanced stages of acute malnutrition ([8]; Section 3.3)—emerged in position, also, to initiate the tolerogenic and non-inflammatory immunological character central to the tolerance model [34,39]. 

The foregoing notwithstanding, blood concentrations of two inflammatory cytokines in particular, TNF-α and IL-6, have been reported over a span of many years to be either elevated or unaffected in cohorts of acutely malnourished children (e.g., [12,99,102,103,104]). Corroborating reports with respect to TNF-α [92] and both TNF-α and IL-6 [105] in the blood of acutely nitrogen-deficient weanling rodents also must be noted. Endotoxemia, a common feature of acute pediatric malnutrition [57], could be a factor in relation to these reports, having been found to trigger an exaggerated TNF-α response when imposed on an adult rodent starvation model [23]. More than this, reports of robust blood concentrations of TGF-β [92] and IL-10 [12,99,104] found together with high levels of IL-6 or TNF-α highlight a need to assess functional balance among cytokines, and to do so in a manner cognizant of the diversity of anti-inflammatory influences exerted by the tolerogenic cytokines (illustrated, for example, by the constellation of direct and indirect mechanisms coming to light with respect to IL-10 [106]). 

It is also important to note that knowledge of the biological roles of IL-6 and TNF-α continues to grow in sophistication. Although generally considered invariantly inflammatory, these cytokines may be best understood as pleiotropic. For example, the IL-6–hepcidin axis promotes hypoferremia, a non-inflammatory defence against extracellular pathogens [107], and this response is reportedly intact in adult rodents subjected to weight loss through caloric deficit [108]. In addition, the cellular source of IL-6 is reported to determine whether the ensuing response (at least in adipose tissue) is inflammatory or non-inflammatory [109]. Similarly, in rodent models, TNF-α appears to constrain type 1-based inflammatory over-reaction to facultative intracellular parasites [110,111], perhaps in part by supporting the formation and maintenance of stable granulomas [111]. TNF-α also is reported to promote the expansion and stability of regulatory T cell populations [112], although this information is limited to tumour models, so its relevance to the pathogenesis of infectious disease remains to be determined. Clearly, TNF-α and IL-6 merit attention in the context of the tolerance model. In Section 6.4, herein, a larger related point is addressed regarding the retention of inflammatory capabilities in the face of acute prepubescent malnutrition. 

### 4.2. The Cytokine Signature of Mononuclear Cells from the Blood and Lymphoid Organs 

An important research front, centered on blood mononuclear cells of acutely malnourished children, continued to grow through the efforts of a group based in Mexico City [98,113,114]. First, using the index of intracellular type 1 (IL-2, IL-18, IL-21 and INF-γ) and type 2 (IL-4 and IL-10) cytokine expression, the group showed that both CD4^+^ and CD8^+^ blood T cell populations from malnourished children exhibited type 2 cytokine polarization [98,113]. In addition, the group reported corroborating results regarding the expression of mRNA for type 1 and type 2 cytokines on the part of the full blood mononuclear cell compartment [98,114], and also found a reduced percentage of CD14^+^ blood mononuclear cells expressing intracellular IL-12 [98]. Other research teams also reported a low percentage of T cells expressing IL-2 [99] and INF-γ [12,99] in blood samples from acutely malnourished children. Further, one group extended the database to include the blood dendritic cell compartment which exhibited (on admission vs. following recovery) diminished constitutive production of IL-12 in vitro together with high-level constitutive production of IL-10 [12]. 

During the same period of years, two studies of young adult rodents subjected to an acute dietary nitrogen deficit yielded broadly similar findings [11,13]. The outcome measures included the expression of several cytokines and two inflammation-associated transcription factors by mononuclear cells from diverse lymphoid organs. In particular, the deficient animals mounted an increased IL-10 response (assessed by blood cytokine concentration) following inflammatory stimulation in vivo [11], and corroborating outcomes were reported on the part of splenic T cells [13] and LPS-responsive mononuclear cells from the spleen [13] and bone marrow [11] when stimulated in vitro. Although malnutrition-associated influences were few and relevance to prepubescent malnutrition is uncertain, the findings appeared, collectively, to reflect a non-inflammatory immune character within both peripheral and primary lymphoid sites. In a concurrent investigation, splenic and nodal T cells from fasted adult mice exhibited marked depression in the production of IL-2 and INF-γ following stimulation in vitro [25], although interpretation for the present purpose is compromised by the absence of data pertaining to non-inflammatory or tolerogenic cytokines. 

The growing database pertaining to cytokine production and blood concentration profiles prompted an investigation to test and refine the tolerance model according to the prediction that acute prepubescent deficits of nitrogen and energy will elicit a shift toward a non-inflammatory identity within characteristically inflammatory lymphoid sites while sustaining the disposition of non-inflammatory sites [40]. Using two weanling models, this investigation addressed the expression of mRNA for IL-10, TGF-β and IL-12 in the spleen, a typically inflammatory lymphoid organ, and in the small intestine, which is characteristically non-inflammatory. These models had been reported previously to exhibit non-inflammatory, tolerogenic blood cytokine profiles [8,35], a systemic predisposition toward type 2 cytokine production [36,38,39] and a type 2 effector/memory T cell character [38], and the outcome of the investigation suggested that the immunological re-shaping associated with both weanling systems centers on typically inflammatory sites. In addition, the findings added TGF-β and IL-12 to the pre-existing list of cytokines (IL-10 [11,36,38,39], IL-4 [38] and INF-γ [38]) for which evidence consistent with the tolerance model was available on the basis of information pertaining to synthesis assessed in vivo. Clearly, the outcome of the investigation refined and extended the tolerance model in a manner indicative of a controlled, systemic attempt to achieve a non-inflammatory and tolerogenic immune character. A precautionary caveat should be emphasized, however, that sustaining the non-inflammatory character of mucosal immune sites must not be overinterpreted as evidence that mature mucosal adaptive defences remain intact. 

### 4.3. Cytokine Assay Techniques Key to the Development of the Tolerance Model 

Two laboratory technologies, each applied to weanling rodents, are noteworthy at this juncture. In the first place, the application of bioassays rather than the understandably popular ELISA appears to have conferred an important benefit in terms of consistent clarity in the estimation of constitutive blood cytokine concentrations [8,35,36,37]. Although seldom openly considered, potentially misleading limitations of the ELISA are a matter of record in relation to the assessment of cytokine concentrations in biological fluids [115,116,117,118]. The ELISA provides estimates restricted to the unbound fraction of cytokine without reference to biological activity, and it appears that this type of assay detects only a small and unrepresentative fraction of the total quantity of biologically active cytokine accessible by bioassay. In particular, direct comparisons with the ELISA point persuasively to the bioassay as more reliable, if validated in terms of specificity, for assessing blood concentrations of IL-10 [117] and TGF-β [118], the tolerogenic cytokines at the heart of the tolerance model [8]. 

The second key assay technology, the in vivo cytokine capture assay [119], provided information directly related to the rate of cytokine production by prepubescent animals during their response to acute forms of malnutrition. The assay revealed robust net systemic production of IL-10, constitutively, even in advanced stages of malnutrition [36,39] and, importantly, permitted the conclusion that the high blood concentrations of this tolerogenic cytokine were not a consequence of a reduced rate of turnover (catabolism). Likewise, the same outcome emerged regarding the production of IL-4 by effector/memory T cells, whereas net production of INF-γ by this cellular compartment declined simultaneously [38]—a comparison between types 1 and 2 cytokines rendered particularly interesting by an assay that permits a view of synthesis, in vivo, largely unimpeded by interference from catabolism. Thus, the in vivo capture assay provided unique direct evidence that, despite a profoundly limited amino acid supply, a non-inflammatory immune character is initiated early in the development of acute prepubescent nitrogen and energy deficits [36] and is sustained into its advanced stages [38,39]. Such an outcome is predicted by the tolerance model but cannot fit within a paradigm of unregulated immunological attrition. 

Enlargement of the aforementioned database is needed both to increase the diversity of experimental systems and to expand the array of tested cytokines. In this context, however, it is relevant to note that the cited information was derived from the application of the bioassay and cytokine capture techniques to two longstanding and validated animal models. The particular merit of the models stems from their demonstrated relevance to severe forms of acute pediatric malnutrition and, in addition, from the database built up over three decades of investigation regarding their immunological characteristics. Although the critical physical and metabolic (particularly hormonal) characteristics of the two animal models have not been summarized in a single, consolidated source, they can be found recorded within the body of research based on the models, and some of these reports are cited herein [8,9,35,36,37,38,39,40,58,59,60,61,72,73,74,75,76,77,79,97,120] because of their particular significance to the development of the tolerance model. A broader treatment of animal modeling for research in pediatric malnutrition [7] also identifies important validating features of the two models. The use of validated and substantively characterized models enhanced the confidence with which the findings yielded by the bioassay and cytokine capture techniques could be interpreted. 

### 4.4. Intervention Studies

Studies reporting responsiveness to endocrine hormones, cytokines and other mediators or mediator-containing fluids (Section 2.1, Section 2.2 and Section 3.2) constituted an important part of the information base leading to the formal declaration of the model in 2006 [8]. Six subsequent intervention studies added to the supportive database, five reporting administration of a hormone or cytokine [9,25,113,121,122] and one reporting an adoptive cell transfer strategy [120]. 

Leptin, having featured in studies leading to the formalization of the model (Section 3.2), continued to be of interest. T cells taken from the blood of acutely malnourished children responded to this hormone in vitro with an increase, evident both constitutively and following mitogen stimulation, in the percentage of cells expressing type 1 cytokines (IL-2 and INF-γ) and a corresponding decrease in the percentage of IL-4- and IL-10-positive elements [113]. The in vitro intervention also restored the capacity of the T cells to express activation markers in response to a mitogenic stimulus. Despite the complication of concurrent infection and the limitations of an in vitro intervention strategy, the investigation stands alone as a chemically defined intervention pertaining to a pediatric population. Subsequently, leptin intervention studies were extended to demonstrate responsiveness on the part of the B cell system in the context of an adult rodent fasting model [121]. In this investigation, administration of leptin either peripherally or centrally (intracerebroventricular) prevented starvation-induced attenuation in B cell development within the bone marrow, and further pharmacological interventions suggested a chain of events mediated through the central nervous system and the hypothalamic–pituitary–adrenal axis. Shortly thereafter, a report based on the same adult rodent model revealed that leptin intervention, either in vitro or in vivo, reinvigorated the production of IL-2 and INF-γ by splenic and nodal effector T cells generated in vitro [25]. Although extrapolation to acute forms of prepubescent malnutrition is hazardous, these findings extended evidence of immunological responsiveness in the face of catabolic malnutrition to include the B cell system and central neuroendocrine regulation [121], and suggested a direct influence on the glucose uptake of activated T cells, and, hence, ongoing responsiveness to “metabolic reprogramming” [25]. 

Two additional interventions each probed responsiveness to a previously untested cytokine. In the first investigation [122], granulocyte colony-stimulating factor elicited a substantial recovery of the blood neutrophil count of adult mice subjected to acute deficits of nitrogen and energy, although the increase in cell numbers was attenuated relative to the response generated by animals fed a complete diet ad libitum. In this piece of work, the intervention was initiated subsequent to the establishment of advanced catabolic malnutrition and evidence of depressed inflammatory competence; therefore, the findings pointed forcefully to the maintenance of immunological plasticity despite advanced wasting disease, albeit in adulthood. The second investigation centered on acutely malnourished weanling mice given exogenous fms-like tyrosine kinase 3 ligand [9], a cytokine both essential and sufficient for the maintenance of dendritic cell compartments [123]. In this piece of work, the intervention prevented depression in the numbers of conventional splenic dendritic cells (CD11c^+^ F4/80^−/low^) despite profound splenic lymphoid atrophy. Moreover, regardless of deepening catabolic disease, the exogenous cytokine supported a vigorous spleen-based cell-mediated adaptive immune response that was, otherwise, profoundly depressed. This report extended a previous piece of work using the same weanling model [120] in which intervention by adoptive transfer of murine dendritic cells at an advanced stage of malnutrition restored the same spleen-based cell-mediated immune response despite ongoing and deepening catabolic disease. 

The body of evidence continues to grow, therefore, pointing to ongoing competence and physiological responsiveness on the part of immune defences, both innate and adaptive, despite profound losses in terms of cellular numbers. Three of the six most recent intervention studies [9,120,122], two of which center on the weanling stage of life [9,120], add to the preceding evidence base (Section 2.2 and Section 3.2) illustrating sustained immune response capabilities in vivo, even in an advanced stage of catabolism, if appropriate regulatory signals are provided. The apparent robustness of cellular compartments known for their rapid turnover, viz. neutrophils [122] and dendritic cells [9], is a small but compelling additional point of detail. 

### 4.5. Metabolic Indices

From its inception [32,33,62] through to the present [7,34], the tolerance model has framed the immunological response to acute prepubescent malnutrition as one component, only, of a larger adaptive attempt. Maintenance of metabolic control, therefore, is anticipated on the basis of the model even into the advanced stages of acute malnutrition. In this connection, sustained and seemingly adaptive metabolic regulation was apparent thirty years ago in terms of fine control over the synthesis of nitrogen-containing compounds as reported, for example, both from studies of acutely nitrogen-deficient weanling and adolescent rats [124,125,126] and from an investigation of children suffering either kwashiorkor or marasmus [127]. In the weanling rat model, expression of genes coding some hepatic proteins increased, e.g., insulin-like growth factor binding protein-1 [124,125] and ferritin [125], while expression of other hepatic proteins either decreased or remained unchanged [125]. Up-regulation of insulin-like growth factor binding protein-1 in this weanling rodent system was, some years later, interpreted by another research team to be part of an adaptive attempt to conserve amino acids for functions of more immediate priority than growth [128]; similarly, reports implicating ferritin in non-inflammatory defence against pathogens [107] point to an adaptation relating more specifically to infectious disease resistance. Subsequent investigations have extended the database beyond the liver, e.g., to include central nervous tissue as indicated from studies of an adolescent rat model of wasting protein deficiency [126]. The latter investigation, like its cited predecessors [124,125], highlighted differential gene expression as a component of the attempt to adapt to an acute nitrogen deficit. This theme is developed more recently and fully elsewhere [128]; moreover, a growing body of complementary information centered on translational regulation [129] may be added to it. The main point to make here, however, is that the cited reports appear incompatible with a paradigm of non-selective decline in the synthesis of amino acid-containing molecules. In this connection, it is important to highlight a modest but compatible database that derives from children suffering severe acute malnutrition and that includes, for example, a report pertaining to the systemic production of cysteinyl leukotrienes [127] together with somewhat more recent estimates of the systemic rate of synthesis of acute-phase proteins and nonessential amino acids [130]. 

The foregoing considerations signaled, even decades ago, a response to acute dietary nitrogen deficiency that includes the controlled apportionment of nitrogen according to a new set of metabolic priorities. This perspective centered on metabolic attempts to adapt to catabolic malnutrition continues to grow and now includes, also, the metabolism associated with acute dietary caloric deficits. As an example, a recent report, albeit based on a young adult mouse model, points to a regulated diversion of energy away from inflammatory responses and toward maintenance of core temperature in response to a catabolic energy deficit [27]. Other recent evidence, also based on a young adult mouse model of acute energy restriction [48], raises the possibility of an entirely unanticipated adaptation in glucose metabolism that would facilitate the maintenance of blood glucose concentrations despite ongoing catabolic malnutrition. Insulin-like growth factor-1 emerged as a key player in this experimental context, provoking lipolysis within white adipose tissue in surprising contradistinction to its familiar anabolic influence. Classical understanding of the physiological actions of some metabolic mediators (and, by extension, other types of mediators) may require revision in the context of acute malnutrition. 

In another research front, bone marrow adipose tissue is reported to accumulate in anorexic humans [131,132], in prepubescent mice subjected to caloric restriction [133] (although without weight loss), in adult mice subjected to a negative energy balance through either caloric restriction [26,131,132,134] or dietary nitrogen deficit [135], and even in diverse species starving in the wild [131]. Marrow adipose expansion also is reported to be a component of the systemic murine inflammatory response to lipopolysaccharide [136] and, of particular note here, was exaggerated in animals subjected, from weaning, to a moderate form of acute nitrogen and micromineral deficit [136]. Concurrent loss of visceral fat reserves has been documented frequently [26,131,132,133], including the case of a rabbit model of caloric restriction in which marrow adipose tissue, although not expanded, was sustained while visceral fat masses declined [134]. The phenomenon appears to be a glucocorticoid-mediated adipogenesis [26,134] that permits the bone marrow to provide a niche for a metabolically quiescent reserve of immunological elements including memory T cells, plasma cells, natural killer cells and regulatory T cells [26]. Despite inevitable physiological costs, e.g., bone fragility [136], the phenomenon appears likely to represent a true adaptive attempt. Certainly, the remodeling appears profound, e.g., up-regulation of close to 4000 genes [26] including a cluster associated with marrow adipogenesis [26,135,136]. Moreover, importantly, a survival benefit against experimental infectious disease is reported [26]. 

Of some interest in the present context, evidence that the remodeled marrow compartment serves as a repository for memory T cells [26] (albeit in a young adult rodent setting) appears complementary to other evidence (Section 3.1) indicating a selective involution of the peripheral effector/memory T cell compartment in acutely malnourished weanlings. On the basis of a recent comment regarding bone marrow adipogenesis and nutritional deprivation [137], it appears that the most sophisticated investigations of the phenomenon continue to center on adult animals and humans subjected either to short-term fasting or to protocols of caloric restriction thought to provide sufficient nitrogen and micronutrients. It will be important to extend commensurate sophistication to dietary contexts that can be considered directly relevant to acute forms of prepubescent malnutrition. Moreover, possible non-immunologically based adaptive benefits also merit research attention. For example, a robust marrow adipose reserve may serve as a necessary source of adiponectin to support systemic homeostatic attempts during caloric deficits [132] and may prove important, also, during recovery from caloric restriction, e.g., as a source of substrates and hormonal support for skeletal renewal [131]. 

## 5. Fitness Test: Application of the Tolerance Model to a Published Clinical Study of Acutely Malnourished Infants and Children 

The tolerance model is in need of testing to gauge its fitness to improve the depth of understanding with respect to immune defences in acute forms of malnutrition. Application of the model to the existing evidence base should be helpful in this regard, and the example that follows illustrates use of the model to re-evaluate the contribution of a published investigation of prepubescent humans. The example provides a basis for optimism that the model can be used to stimulate insights and questions, and ultimately advancements in understanding, that did not—and could not—arise through the classic lens of immunological attrition. Further such analyses appear worthwhile. 

A report regarding a cohort of Ugandan infants and children suffering severe acute malnutrition [104] is noteworthy for two reasons. First, it provides an exceptionally broad coverage of the blood metabolome and, secondly, it provides a sequential assessment of the impact on the metabolome achieved first by inpatient care (after which the participants remained severely wasted, although medically stabilized and free of edema) and subsequently by outpatient support. In this investigation, classic endocrine hormones and adipokines together with nutrient and metabolite indices presented collectively and, for the most part, individually according to established expectations. By contrast, blood cytokine levels were largely unaffected except that, regardless of the form of malnutrition, high blood concentrations of three type 1 cytokines (IL-2, IL-6 and TNF-α) proved independently predictive of inpatient mortality. In addition, the blood IL-10 levels of the cohort that survived to reach the stage of outpatient treatment (stable but still severely wasted) remained high relative to the levels exhibited following rehabilitation. The tolerance model suggests and permits an interpretation in which, despite infectious challenge, a failure to down-regulate tissue fluid concentrations of inflammatory cytokines reflects a life-threatening immunological rigidity (inability to adapt). In this case, therefore, the application of the tolerance model yields a testable interpretation of a data set on which an attrition-centered paradigm can shed little light. Parenthetically, others have alluded to the same unconventional interpretation inasmuch as high blood concentrations of inflammatory cytokines were predictive of mortality, but unrelated to pathogen load, in a cohort of children under five years of age suffering severe acute malnutrition [138]. The open question of inflammation in the acute malnutrition syndrome is addressed further in Section 6.4. 

## 6. The Tolerance Model: Robustness and Prospects

Applying the tolerance model to existing information should prove productive, but it must be kept in mind that the model remains a proposition. An assessment follows regarding the speculation that the systemic catabolism of acute malnutrition, in fact, creates a threat of inflammatory autoimmune pathologies (Section 6.1). In addition, the extent is examined to which the model is bound by some presuppositions generally associated with it (Section 6.2, Section 6.3, Section 6.4). Finally, the potential of the model is illustrated for growth and development within the purview of its host discipline of immunobiology (Section 6.5). As a subordinate point arising from this section, the substance of the tolerance model proposition is likely to be better represented going forward, and its usefulness enhanced, by extending beyond the narrow confines of immune tolerance to center attention on non-inflammatory immune character. 

### 6.1. Does Acute Pediatric Malnutrition Impose a Risk of Autoimmune Disease? 

According to the tolerance model, the primary benefit of fostering a tolerogenic form of immune competence is to minimize the risk of autoimmune inflammation pursuant to the catabolic release of self-antigens normally hidden from immunological view [7,8,32,34,36,37,39]. It has been rightly pointed out, therefore, that one would anticipate autoimmune pathologies to be at least an occasional part of the childhood malnutrition syndrome, but that no such manifestations have been identified thus far [5]. Evidence is scant, at best, linking acute prepubescent malnutrition either with autoimmune disease or with tolerogenic cellular elements. 

Twenty years ago, epidemiological information was cited to the effect that autoimmune disease appears to be more prevalent in settings described as “affluent and more developed societies” than in settings described as “less developed” [30]. Although no firm connection can be made with diet or energy consumption on the basis of this type of information, animal-based investigations were interpreted similarly as showing that diet, including caloric intake, can play a meaningful role in the initiation and progression of autoimmune pathologies [30]. A few years later, caloric restriction sufficient to cause weight loss and depressed cell-mediated inflammatory competence was reported to prevent initiation of autoimmune encephalomyelitis in adult rats, perhaps “through impaired INF-γ production” [139]. Subsequently, in a brief summary of animal-based evidence, a similar suggestion was offered that “calorie-restriction and fasting prevent autoimmunity, likely by decreasing T cell responses and inflammatory cytokine production” [140]. 

The foregoing speculations are enticing in relation to the tolerance model, but they are based entirely on association. Moreover, others [141] point out plausible alternative possibilities that do not invoke cytokine-based conjecture, notably the inhibition of damaging oxidative reactions. It should be noted, also, that the animal-derived evidence is based, with rare exceptions (e.g., [139]), on rodent dietary models that cause stunting, not wasting, when imposed on weanling or adolescent animals, and disease-associated indices are then assessed after the animals have reached adulthood. The relevance of such experimental systems to acute forms of prepubescent malnutrition, therefore, appears slight and easily overinterpreted. Importantly, equal restraint is needed, for the same reasons, when attempting to connect the tolerance model with information, summarized recently [142], pointing to the protective benefits of various forms of dietary restriction in relation to clinical manifestations of multiple sclerosis in humans. Ultimately, documentation from acute human pediatric malnutrition is needed, although the classic problem of survivor bias (suggested by a disproportionately high risk of mortality reported among those least able to control inflammation (Section 5)) may render such information particularly difficult to acquire. A worthy speculation suggests autoimmune roots on the part of some ill-understood clinical presentations such as the dermatoses often seen in edematous forms of acute pediatric malnutrition [5]. 

Self-tolerance requires an array of cellular elements [143], and the tolerance model predicts sustained or even up-regulated activity on their part. In this connection, a starvation protocol reduced the cell-mediated inflammatory competence of adult mice while increasing the proportion of lymph node CD4^+^ T cells expressing Foxp3 [144], a transcription factor identifying a particularly important subset of regulatory T cells within the peripheral tolerance arsenal [145]. Similarly, adult mice subjected to intermittent fasting exhibited a reduced proportion of IL-17^+^ T cells together with an increased proportion of Foxp3^+^ T cells in the small intestinal lamina propria [146]. Meanwhile, in a related investigation performed as an intentional test of the tolerance model [40], weanling mice were subjected to acute forms of nitrogen and energy deficit that elicit involution of T cell compartments and depression in cell-mediated inflammatory competence. The animals, nevertheless, sustained their expression of Foxp3 mRNA in both the spleen and the small intestine, although the cellular origin of the transcription factor (perhaps not unique to regulatory T cells [147]) was not identified. Although consistent with predictions of the tolerance model, clearly, the evidence pertaining to tolerogenic cellular elements is meagre, being limited to animal studies (only one of which is directly relevant to the prepubescent stage of life), one aspect of peripheral tolerance, and expression of a cellular marker, rather than an assessment of function. 

### 6.2. Is a High Risk of Autoimmune Pathologies Essential to the Tolerance Model?

As suggested more than 25 years ago [29], and reiterated more recently [11], attenuation of inflammatory vigour consequent to catabolic malnutrition may bring with it a benefit in terms of minimizing infection-related collateral damage. This reasonable conjecture clearly intersects with the tolerance model, with neither proposition excluding the other, although the purported benefit was expressed as an epiphenomenon rather than as a consequence of an adaptive attempt. More recently, others [12,27] have proposed benefits centered on the concept of adaptation—hence, compatible with the core theme of the tolerance model—while requiring no reference to a risk of autoimmune disease. The possible “survival advantage of anergy in the face of severe malnutrition” [12] presumably means a metabolically determined advantage in terms of energetics. Similarly, the “disease tolerance” noted recently in a rodent model [27] appeared to reflect a controlled diversion of energy from costly inflammatory defence toward maintenance of homeothermy in the face of limited resources of energy. The important point here is that the tolerance model accommodates diverse potential benefits that may accompany a non-inflammatory immune defence strategy. The model centers on the concept of a regulated adaptive attempt; hence, its core value does not depend on the verification of the notion that acute malnutrition creates a risk of autoimmune pathologies. 

A brief consideration of the “disease tolerance” concept may be instructive in the present context. Disease tolerance is a non-immunological defence strategy, increasingly acknowledged in recent years, that limits damage to an infected host without exerting a direct negative influence on the infecting pathogen [27,107,148]. Its underlying mechanisms are poorly defined, but are emerging in the form of a metabolic network that may carry adaptive potency in relation to infectious challenge comparable to the recognized importance of classic immune resistance [148]. With the concept of disease tolerance in mind, the energetic cost of immune defence against infection was recently explored in an investigation suggesting that “energy conserving hypometabolic states”, e.g. caloric restriction, respond to an infectious challenge by directing limited energy resources away from immune defences and toward maintenance functions, notably thermoregulation in the case of an endotoxic provocation [27]. In this investigation, adult mice were subjected to an energy deficit by means of alternate-day fasting and, when subsequently challenged with a bacterial pathogen, were judged to exhibit “disease tolerance” (based on survival together with hepatic and splenic pathogen counts) rather than immunologically based disease resistance. The relevance of an adult alternate-day fasting model to acute prepubescent malnutrition remains to be seen. As a coordinated metabolic effort to direct limited energy resources away from costly inflammatory defences, this adaptive and defensive strategy fits comfortably within the purview of the tolerance model and could either add to, or supplant, the original notion of protection against autoimmune pathologies. 

### 6.3. Is an Emphasis on Secondary Immune Deficiency Essential to the Tolerance Model?

For many decades, prepubescent malnutrition, at least in its severe and acute forms, has been widely considered to elicit diverse clinically significant deficiencies in immune defences (e.g., [3,4,5,6,32,56,57,149]). In view of the weight of evidence and opinion, it may be worthwhile to note that the accepted cyclic association among malnutrition, depressed inflammatory capacities and infection does not imply unidirectional causation between any pair of these factors, nor is there a clear basis for identifying any of the three factors as the dominant point of initiation for the cycle in the human pediatric setting [19]. In fact, from a clinical perspective, acute pediatric deficits of nitrogen and energy, even in severe forms, may exert quite insubstantial direct effects on inflammatory immune competence, at least in some settings [12,19]. Moreover, the possibility that primary immune deficiencies may play an important role in the cycle [19] is plausible but largely unexplored. Importantly for the present purpose, however, de-emphasizing or even dismissing the factor of direct secondary immune deficiency presents no challenge to the tolerance model. The central tenet of the model, viz. sustained metabolic and immunological adaptive capacities despite profound catabolic pathology, rests independently of any assumptions regarding the primacy of nutritional deficits, or even infection, to the cycle of malnutrition and infectious disease. 

### 6.4. Can the Tolerance Model Accommodate Evidence of an Inflammatory Response?

Depressed inflammatory and febrile competence is a long-recognized clinical feature of acute pediatric and experimental weanling malnutrition [6,7,57,150,151]. Moreover, a significant body of information testifies to a non-inflammatory immunological character in terms of cytokine-producing predisposition (Section 3.3, Section 4.2 and Section 4.3; [3,10,15,18]) and blood concentrations of the potently tolerogenic triad of cortisol (or the rodential equivalent, corticosterone), IL-10 and TGF-β (Section 2.1, Section 3.2, Section 3.3 and Section 4.1). Nevertheless, evidence of an inflammatory capability within the acute malnutrition syndrome must not be overlooked, as is demonstrated persuasively by recent reviews of pediatric malnutrition and immune defences [5,152]. This evidence takes the form, particularly, of blood biochemical and immunological indices recognized as elements of an inflammatory microenvironment [5,12,92,99,102,103,104,105,138], a point outlined in Section 4.1, herein, with emphasis on concentrations of TNF-α and IL-6. 

A more insightful and nuanced grasp is needed regarding the inflammatory response in acute forms of pediatric malnutrition. In this connection, endotoxemia is a common feature of acute prepubescent malnutrition [57], and endotoxemic inflammation appeared likely to underlie the cytopenic and anti-inflammatory (IL-10-producing) blood dendritic cell compartment that was predictive of anergy in a cohort of children suffering severe acute malnutrition [12]. Both human- and animal-based studies suggest a sequence in which malnutrition-induced intestinal dysbiosis and epithelial barrier compromise precede endotoxemia and indications of systemic inflammation [3,105,153]. An interesting suggestion is that endotoxin tolerance is sustained even by severely malnourished children, thereby “blunting the acute-phase response and the production of inflammatory mediators”, albeit variably, depending on the particulars of intestinal dysbiosis and epithelial insult [15]. Endotoxin tolerance reflects an adaptive capacity centered on IL-10 and TGF-β [154]; hence, this proposition invites pursuit both on its own merits and in the context of the tolerance model, although reconciliation is needed with a report of heightened lethal endotoxin sensitivity on the part of adult rodents subjected to short-term starvation [23]. In any case, the tolerance model invokes regulated limitation, not elimination, of the inflammatory response to infection as an appropriate, perhaps even necessary (Section 5), adaptive capability in the context of acute pediatric malnutrition. Thus, inflammatory capabilities (requiring ongoing down-regulation) can be anticipated within the framework of the model. 

Recently, mice subjected to acute malnutrition from weaning through adolescence were reported to present an amplified inflammatory response within the bone marrow in terms of cytokine profile and the influx of inflammatory monocytes [136]. In turn, this exaggerated response was implicated in the heightened adipogenic differentiation observed on the part of mesenchymal stem cells within the bone marrow of the malnourished animals. Although the investigation was confined to inflammatory indices and the marrow, its outcomes nonetheless raise the intriguing possibility of an attempt to sustain, in a tissue-specific manner, a complement of inflammatory capacities needed to promote the immunological (and other) benefits ascribed to marrow adipogenesis in the context of acute malnutrition (Section 4.5). The larger point remains, however, that the tolerance model, being based on a thesis of regulated immune defence capabilities (in contrast to unregulated attrition), accommodates the persistence of a full complement of immune defence functions in the face of catabolic forms of pediatric malnutrition. 

### 6.5. Can the Tolerance Model Co-Evolve with Immunobiology? 

As a subdiscipline of immunobiology, the study of malnutrition-associated immune defence requires a paradigm that can grow with the larger field. In view of the prominent theme of immune regulation within the tolerance model, it is instructive to consider the model in juxtaposition with emerging insights regarding regulatory immunobiology. 

Peripheral anti-inflammatory suppressor cells are recognized as critical to immune regulation and self-tolerance [143,145,155,156]. In this respect, progress has been particularly rapid in relation to CD4^+^Foxp3^+^ T cells [143], and information pertaining to these elements has begun to enrich the tolerance model (Section 6.1). Wide-ranging regulatory activity also can be ascribed to diverse subsets of B cells both in health and in a plethora of inflammatory diseases including autoimmune pathologies, infections and allergic conditions [155]. This research front continues to develop in fundamental ways. For example, evidence of innate regulatory B cells has been added recently to the preceding catalog of information centered on antigen-specific subsets [156]. In the present context, it is of interest that regulatory B cells appear to act by way of IL-10 [155,156] and TGF-β [155], the tolerogenic and non-inflammatory cytokine dyad at the heart of the current iteration of the tolerance model ([7,9,34], Section 4.1 and Section 4.2). In addition, some subsets of regulatory B cells produce suppressive IgM and IgG immunoglobulins [155], a point of interest here in view of the longstanding observation [5,6,57] that blood concentrations of these classes of immunoglobulin, much of which must be a polyreactive natural antibody [6], are unaffected even in the most severe forms of acute pediatric malnutrition. The inclusion of the developing knowledge base pertaining to anti-inflammatory peripheral regulatory cells, in all their diversity (i.e., extending beyond lymphoid lineages [143,155]), is necessary for the healthy growth of the tolerance model and is in character with it. 

## 7. Concluding Perspective

The central tenet of the tolerance model—a regulated and adaptive modification of immune defences in the face of acute forms of prepubescent malnutrition—was first proposed more than thirty years ago [32,33], and the model was put forward formally in 2006 [8] as a paradigm with a disperse but assertive evidence base. The designation of the model centered on self-tolerance mainly because of the blood cytokine profile emphasizing the tolerogenic dyad of IL-10 and TGF-β (Section 3.3). Although there continues to be no evidence that acute malnutrition increases the risk of autoimmune complications (consequent to catabolic release of self-antigens) and the problem of survivor bias may render acquisition of such information difficult (Section 6.1), clinical characteristics suggestive of autoimmune risk in edematous pediatric malnutrition have been noted [5] and appear worthy of pursuit. Independently of speculations centered on autoimmune inflammations, the concept of infectious disease tolerance [27,107,148] positions comfortably within the tolerance model (Section 6.2). Likewise, endotoxin tolerance merits attention within the context of acute prepubescent malnutrition ([15], Section 6.4) and could add important substance to the model. Consequently, either infectious disease tolerance or endotoxin tolerance (or both) could reasonably shift attention toward infectious agents and away from self-antigens. In hindsight, a designation such as “non-inflammatory competence model” would have captured the essence of the proposition more robustly than the title “tolerance model”. 

The most conspicuous and distinguishing implication of the tolerance model is that the immunological characteristics of acute prepubescent malnutrition confer physiological benefits and, in addition, reflect a larger adaptive metabolic response. Hints along each of these lines can be gleaned from recent animal-based studies that suggest benefits both in terms of energy-efficient preservation of diverse immune defence elements ([26], Section 4.5) and in terms of systemic energy conservation ([27], Section 6.2). These are attractive possibilities, but it is important to recognize that the adaptive nature of malnutrition-associated immune defence characteristics remains a matter of conjecture that arose, as outlined in Section 2.1, from the point of view, now paramount to the burgeoning discipline of immunometabolism [137,157], that immune defences are best understood as components of systemic metabolism. Within the field of immunometabolism, a spectrum has emerged ranging from the inflammatory manifestations of obesity to the non-inflammatory adaptations of modest caloric deficits that provoke “a negative energy balance without malnutrition” [137]. In effect, the tolerance model extends this concept, although preceding it chronologically, to include acute forms of malnutrition, particularly during, but by no means limited to, the prepubescent stages of life. 

Connecting the immunological characteristics of malnutrition with a demonstrated adaptive benefit is a matter of some urgency to the development of the tolerance model and must be expected to require animal-based investigations demonstrably relevant to acute pediatric malnutrition. The need for improved attention to design and modeling in the experimental study of acute prepubescent malnutrition is documented [3,7], and the potential of a consistent effort in this regard is illustrated by the research background of the tolerance model which was first both tentatively proposed [32,33] and enunciated formally [8] within the context of a decades-long commitment to a research program centered on the immunological characteristics of two validated weanling rodent models ([7], Section 4.3). The same research program ultimately yielded an information base germane to potential medical applications of the tolerance model canon by directing attention away from the classic T cell-centric focus and toward dendritic cells and antigen presentation within the setting of pediatric malnutrition-associated immune depression [7,9]. 

The potential of immunoenhancement to augment the management of acute pediatric malnutrition has been voiced for many years [158,159,160,161,162,163], and the tolerance model provides a basis for optimism in this respect [7,32]. The model posits that sufficient immunological plasticity is sustained into the advanced stages of catabolic malnutrition to permit the restoration of controlled inflammatory competence without the prerequisite of stabilizing nitrogen and energy balance. Evidence consistent with this prediction derives from animal-based intervention studies beginning with the surgical strategy of adrenalectomy and spanning three endocrine hormones (triiodothyronine, leptin and a glucocorticoid), five cytokines (IL-1, granulocyte-macrophage colony-stimulating factor, macrophage colony-stimulating factor, granulocyte colony-stimulating factor and fms-like tyrosine kinase 3 ligand), a purified and chemically defined β(1-3) glucan (lentinan), a thymic hormone-containing extract, an endogenous pyrogen-containing preparation, a probiotic, and adoptively transferred elements of a conventional dendritic cell line (Section 2.1, Section 2.2, Section 3.2 and Section 4.4). 

**Figure 4:**
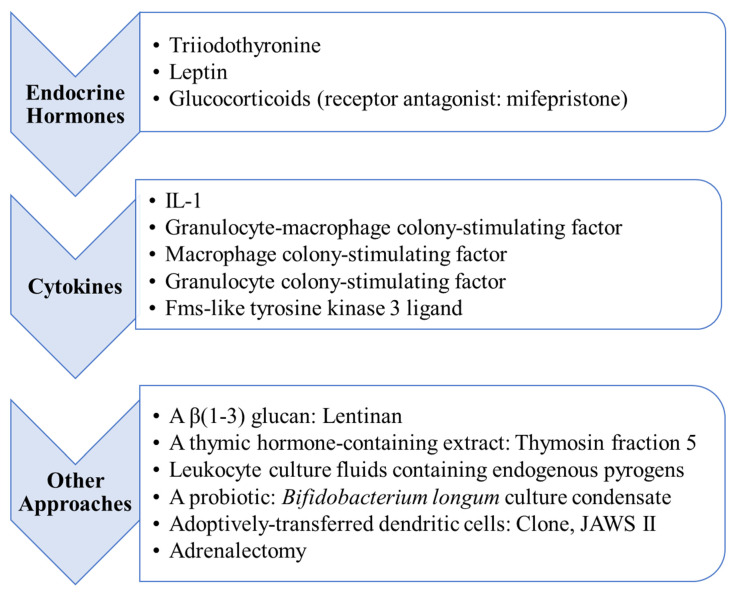
Summary of interventions demonstrating sustained immunologic plasticity in diverse forms of acute experimental nitrogen and energy deficiencies.

Leptin stands alone in having the support of an intervention study involving malnourished children [113], although the investigation was restricted by ethical constraints to an in vitro intervention. On the other hand, the evidence from animal studies regarding leptin is limited to a two-day adult rodent starvation model. The need for improved sophistication in animal modeling cannot be over-emphasized [3,7], but the collective body of evidence from intervention studies (Figure 4) is at least arresting as it spans six decades, derives from fifteen independent research teams and interventions and represents eleven distinct animal models of severe catabolic malnutrition, of which seven center on the weanling stage of life and two more address older prepubescent stages. The important common outcome of the intervention studies is the demonstration of sustained responsiveness, in terms of immunological indices and infectious disease resistance, when mediators of inflammatory immune competence were administered in the face of an unabated negative nitrogen and energy balance. Collectively, the interventions provide proof-of-concept that immunological adaptability, and, hence, an opportunity for clinical management, persists even prior to the clinical stabilization of severe acute malnutrition. Emphasis is added to this point both by the rapidity with which immune defence indices and capacities were restored in investigations with weanlings [64,82,120] and older prepubescent animals [63,65] in which interventions were initiated during advanced stages of weight loss and by the consistent finding that responses to the interventions were achieved in these studies regardless of deepening catabolic malnutrition. From a clinical perspective, therefore, the tolerance model offers hope in the bleak setting of severe acute childhood malnutrition, and the evidence provided by intervention studies, alone, justifies the pursuit of the proposition. 

Articulation of the tolerance model [8] was motivated, in significant measure, by a desire to facilitate the revitalization of a field which, as discussed elsewhere [7], was (and remains) in need of a fresh paradigm. Whereas a model of unregulated immunological attrition was sufficient to energize the early stages of inquiry regarding the impact of catabolic prepubescent malnutrition on immune defence, such a paradigm lacks the sophistication necessary to carry the field and can only point, ultimately, to discouraging limitations in clinical management opportunities [7]. Evidence has been cited herein that, as acute malnutrition progresses, immunological adaptability is sustained (Section 2.1, Section 2.2, Section 3.2 and Section 4.4), thereby permitting the remodeling of immune defences toward a non-inflammatory form of immune competence ([26,27], Section 3.3, Section 4.1 and Section 4.2). Parenthetically, it is of interest to note that the distinctly different physiological challenge of pregnancy is widely considered, also, to elicit an adaptive response in the form of a shift toward type-2 non-inflammatory immune defence competence [164]. The proposition of the tolerance model in this regard, therefore, is by no means an isolated physiological eccentricity. Evidence is provided, also, that the application of the tolerance model to the existing information base will yield new insights regarding severe acute malnutrition in childhood (Section 5 and Section 6.4). Finally, because the model possesses the robustness to grow with its parent field of immunobiology (Section 6.5), it may be realistic to hope that it can stimulate questions of sufficient depth and consequence to invigorate the research effort centered on immune defences and acute pediatric malnutrition.
